# Percutaneous Treatment of Venous Erectile Dysfunction

**DOI:** 10.3389/fcvm.2020.626943

**Published:** 2021-02-02

**Authors:** Hanno Hoppe, Nicholas Diehm

**Affiliations:** ^1^SwissIntervention Microtherapy Center, Bern, Switzerland; ^2^University of Bern, Bern, Switzerland; ^3^Vascular Institute Central Switzerland, Aarau, Switzerland

**Keywords:** erectile dysfunction (ED), impotence, veno occlusive disease, venous leak, embolization

## Abstract

Erectile dysfunction is a defined as recurring inability to achieve and maintain satisfactory erection for sexual intercourse associated with relevant life impairment. The underlying etiologies may be manifold and complex. Currently, vascular etiologies are highly prevalent especially amongst elderly men. Of special interest, especially venogenic causes are of increasing relevance. Therapeutic options comprise risk factor modification, pharmacotherapy, surgical treatment, and endovascular treatment. Especially endovascular treatment options have recently increased in popularity including transcatheter embolization procedures for veno-occlusive dysfunction.

## Introduction

The definition of erectile dysfunction or impotence is described as recurring inability to achieve and maintain satisfactory erection for sexual intercourse (1). The etiologies of erectile dysfunction may be manifold and complex (2). Potential underlying etiologies can be either psychogenic or organic. Psychogenic etiologies of erectile dysfunction may include depression, anxiety, and partner-related difficulties. On the other hand, organic erectile dysfunction can be due to vasculogenic, endocrinologic, neurogenic, iatrogenic, and structural components.

Regarding vascular etiologies of erectile dysfunction, a regular erectile function depends on adequate arterial inflow as well as venous outflow occlusion. Both arterial inflow stenosis or impaired venous outflow occlusion compromises filling of the corporeal bodies. Either problem, namely compromised arterial inflow or venous leakage, may result in vasculogenic erectile dysfunction (3).

In younger patients with arteriogenic erectile dysfunctions it was previously reported that blunt pelvic trauma may cause endothelial dysfunction with reactive atherosclerosis resulting in focal arterial stenosis of the distal internal pudendal artery passing through Alcock's canal. In this location the internal pudendal artery is highly susceptible to blunt mechanical trauma due to compression against the ischio-pubic ramus (4). On the contrary, older patients with arteriogenic erectile dysfunction tend to have more diffuse atherosclerotic disease. In this patient population, erectile dysfunction may be secondary to potential plaque formation in all arterial vasculature involved with penile tumescence (5).

In patients with venogenic erectile dysfunction veno-occlusive dysfunction was recognized as underlying cause in most cases (Box 1). Veno-occlusive dysfunction may result from age or injury related changes to the tunica albuginea, cavernosal smooth muscle dysfunction from structural alterations, excessive adrenergic input or from shunts created during priapism episodes and subsequent repair (6, 7). Although the underlying mechanisms of veno-occlusive dysfunction are not yet fully understood, several conditions such as age, diabetes, prostatectomy, pelvic radiation, and androgen deprivation therapy appear to be potential risk factors (8). Of interest, the average age of patients with venogenic erectile dysfunction was found to be significantly lower (51 years) than that of patients with arteriogenic erectile dysfunction (59 years) (9).

Box 1 **Vascular etiologies of erectile dysfunction.**

Vascular etiologies of erectile dysfunction are highly prevalent increasing with age. Especially venogenic causes are of increasing relevance.

Most studies have shown an increase in the prevalence of vasculogenic erectile dysfunction with aging (7, 10). Therefore, Wespes et al. hypothesize that the erectile dysfunction of aging may be the result of atherosclerosis-induced cavernosal ischaemia leading to cavernosal fibrosis and subsequent veno-occlusive dysfunction concluding that erectile dysfunction due to aging appears to be a slowly progressive disorder and that it may be wise for patients to seek medical intervention earlier rather than later (11).

Therapeutic options comprise risk factor modification, pharmacotherapy, surgical treatment, and endovascular treatment (9). Especially endovascular treatment options have recently increased in popularity including transcatheter embolization procedures for veno-occlusive dysfunction. This chapter outlines diagnosis and endovascular treatment of erectile dysfunction due to venous leakage including study results eliding a systematic analysis.

## Diagnosis

Pre and post treatment patient's erectile function should be assessed using the International Index of Erectile Function questionnaire (IIEF-6) score (12). Prior to treatment patients should have clinical evaluation to rule out potentially underlying psychogenic causes (Box 2).

Box 2 **Diagnosis of venogenic erectile dysfunction.**

In patients with suspected veno-occlusive dysfunction on Duplex ultrasound, CT cavernosography should be performed for morphological depiction of venous leaks in absence of sufficient penile rigidity.

This is crucial for adequate patient selection for endovascular treatment.

### Duplex Ultrasound

Patients with organic erectile dysfunction should be examined with color Doppler flow analysis using direct pharmacological stimulation with an intracavernosal injection of 10–20 μg prostaglandin E_1_. Diagnostic criteria for veno-occlusive dysfunction are a high systolic flow rate >25 cm/s (peak systolic velocity) and a persistent end-diastolic velocity of >5 cm/s 15 min post-injection (rigid phase) with a resistive index <0.75 (7).

### Cavernosometry

In patients with suspected veno-occlusive dysfunction based on duplex ultrasound (e.g., high end-diastolic velocities), dynamic cavernosometry, and cavernosography (DICC) should be performed as accepted gold-standard for diagnostics (13). Dynamic cavernosometry requires pharmacological stimulation with intracavernosal injection of prostaglandin E_1_ in order to obtain a rigid erection. Subsequently, fluid is injected into the penis using certain rates and pressures in order to obtain information about changes of pressure within the corpora cavernosum during penile erection. For cavernosography, contrast medium is injected to visualize potential venous leakage on venogram during penile erection.

### Cavernosography

The diagnosis veno-occlusive disease may be confirmed by dynamic pharmacologic cavernosography. Therefore, a 21-G needle is inserted into the corpora following intracavernosal injection of 20 μg prostaglandin E_1_. After ~30 min intracorporal power injection of 120 ml of a 50% solution of non-ionic contrast agent is performed with a flow rate of 2 ml/s. On cavernosography, potential sites of venous leakage can be identified: Deep dorsal vein, cavernosal veins, internal pudendal veins, periprostatic plexus, external pudendal veins, or iliac veins. Diagnosis confirmation of veno-occlusive dysfunction includes demonstration of venous leakage on cavernosography and associated absence of sufficient penile rigidity.

### Computed Tomography Cavernosography

**Figure 1:**
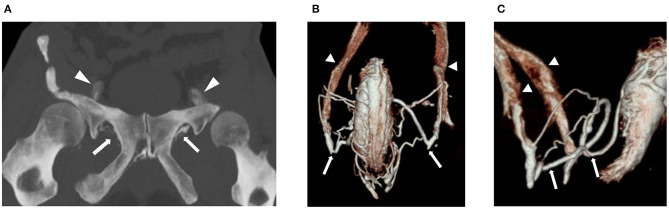
55-year old man with erectile dysfunction due to veno-occlusive disease. Patient is non-responder to PDE-5-inhibitor and Caverject. **(A)** Contrast enhanced CT cavernosography (coronal maximum intensity projection) demonstrates bilateral venous leaks via pudendal veins (arrows) draining into iliohypogastric veins (arrowheads). **(B,C)** Contrast enhanced CT cavernosography (volume rendering in coronal **(B)** and sagittal **(C)** views) demonstrating bilateral venous leaks via pudendal veins (arrows) draining into iliohypogastric veins (arrowheads).

In case of positive findings for veno-occlusive disease in Duplex ultrasound additional computed tomography (CT) cavernosography should be performed to morphologically demonstrate leakage of penile veins. CT images, especially reformatted data using multiplanar reconstruction (MPR), maximum intensity projection (MIP), and volume rendering (VR) techniques, in order to depict details of venous leakage (Figure 1) including penile veins, origin of the crural vein, periprostatic venous plexus, pudendal veins, and drainage into iliac or femoral veins. Penile venous drainage can be divided into three groups: superficial veins, intermediate veins, and deep veins (14). The types of deep dorsal vein and periprostatic venous plexus as well as the origin of crural veins should be carefully assessed providing important information for surgical or endovascular treatment. Accordingly, CT cavernosography may be beneficial in terms of diagnosis of veno-occlusive disease and patient selection for either surgical or endovascular management.

CT-cavernosography is performed following intracavernosal injection of 20 μg prostaglandin E_1_. Thirty min post-injection a 7-G needle is inserted at the dorsal side of the corpora cavernosum and injection of 30–60 ml of 30% saline-diluted non-ionic iodinated contrast medium (320 mg ml^−1^) is performed with an infusion velocity of 6–180 ml min^−1^ (15). Recommended CT parameters are as follows: 64 × 0.625 mm collimation, gantry rotation time 0.75 s, time resolution 30 ms, pitch factor 0.984. The tube voltage is 80 kV, and the tube current-time is 51–90 mAs. Scanning range extends from the upper brim of the true pelvis to the most distant level of the penis. The data constructive section thickness is 1 mm with a reconstruction increment of 1 mm for post-processing.

## Endovascular Treatment

In case of positively confirmed venous-occlusive dysfunction including its morphological demonstration on CT cavernosography, the treatment strategy is occlusion of venous leaks (Box 3). Surgical therapy consists of deep dorsal vein ligation and additional ligation of potential collaterals. However, surgical treatment is rather invasive and usually needs to be performed in an operation room under general anesthesia. Not very encouraging, long-term success rates of surgical ligation of the deep dorsal vein and its collaterals are reported to be ~ 25% (16, 17).

Box 3 **Endovascular treatment of venogenic erectile dysfunction.**

Endovascular treatment can be performed using either antegrade access via the deep dorsal penile vein, which should be the preferential route, or using a retrograde transfemoral venous approach.

For venous leak embolization a mixture of Histoacryl and Lipiodol is used by the majority of physicians. In addition, fibered coils may be used to prevent progression of glue from veins with fast outflow.

Surgical ligation is rather invasive and long-term success rates are not very encouraging. However, surgical exposure of the deep dorsal penile vein may be beneficial to enable venous access prior to transcatheter embolization in selected patients.

### Antegrade Approach via Deep Dorsal Penile Vein

**Figure 2:**
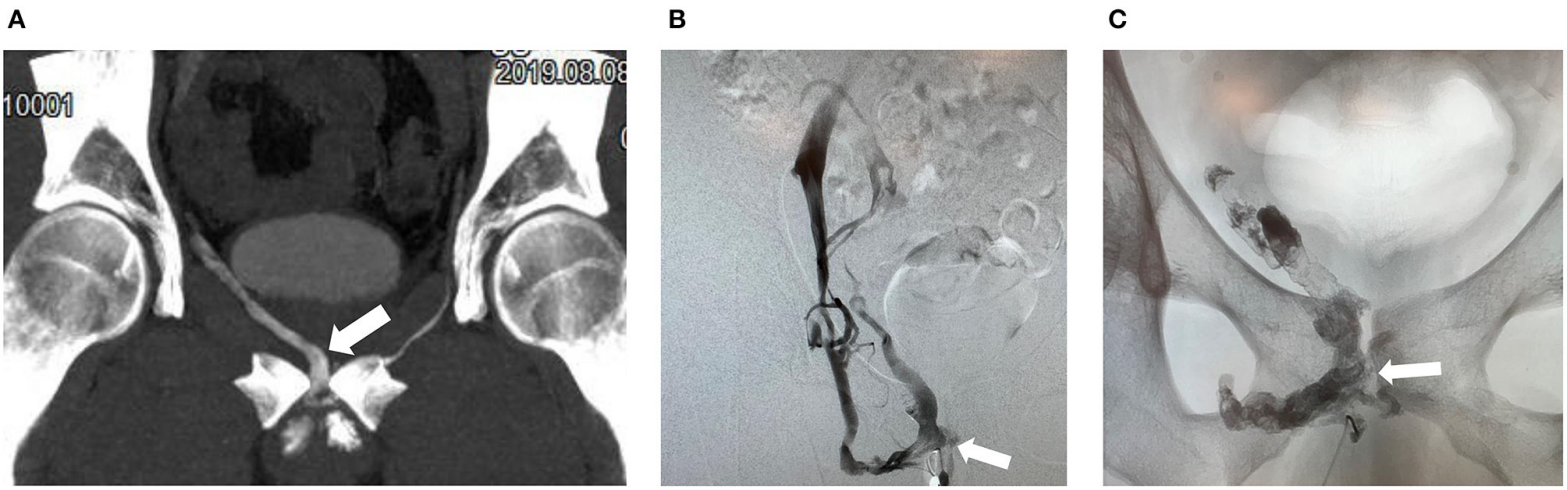
50-year old man with erectile dysfunction due to veno-occlusive disease. **(A)** Contrast enhanced CT cavernosography (coronal maximum intensity projection) demonstrates right sided venous leak via periprostatic veins (arrow). **(B)** Cavernosography with access via the deep dorsal vein and injection of contrast medium demonstrates right sided venous leaks predominatentely via periprostatic veins (arrow). **(C)** Post embolization of periprostatic veins (arrow) using N-butyl-2-cyanoacrylate mixed with Lipiodol demonstrating radiopaque intravenous embolization material (arrow).

The aim of endovascular treatment is sufficient embolization of periprostatic veins and related efferent veins such as for instance internal or external pudendal veins. The antegrade approach is reported to be a safe and efficient endovascular treatment method (7). The procedure is usually performed in an angiosuite. The patient is prepared and draped in the supine position. Following the local subcutaneous administration of lidocaine 2% ultrasound-guide puncture of deep dorsal vein is performed using a 20-G micropuncture set (e.g., Cook Inc., Bloomington, Indiana, U.S.A.) with a 0.018-inch guide wire and 4-F introducer. Subsequently, a combination of a 4-F diagnostic catheter (e.g., KMP, Cook Inc., Bloomington, Indiana, U.S.A.) and a 0.035-inch glidewire (e.g., Terumo, Tokio, Japan) may be used for catheterizing veins more selectively under fluoroscopic guidance. Following a diagnostic venogram embolization is performed using N-butyl-2-cyanoacrylate (Histoacryl by Braun, Melsungen, Germany) and ethiodized oil (Lipiodol by Guerbet, Zurich, Switzerland) mixed in 1:1–1:3 ratios depending on proximity, size and extent of venous leaks (average amount of N-butyl cyanoacrylate ~ 1–3 ml) under Valsalva maneuver and continuous fluoroscopic control in neutral projection with optional oblique projection if necessary to prevent inadvertent progression of embolization materials with non-target embolization (Figure 2). Catheters need to be flushed using 5% glucose solution in order to preserve catheter patency and prevent its inadvertent adhesion to the vessel wall. In addition, fibered coils may be used to prevent progression of glue from veins with fast outflow e.g., into femoral or iliac veins. Oversizing of coils up to 50% of the actual vein diameter is recommend in order to prevent inadvertent coil migration. Periinterventional sedation and pain medication may be necessary.

### Retrograde Transfemoral Venous Approach

Alternatively, transcatheter embolization can be performed using a transfemoral venous approach via internal iliac veins selecting internal pudendal veins and periprostatic venous plexus as previously reported by Aschenbach et al. (18). Embolization of venous leaks was performed via a transfemoral venous access route using a mixture of Histoacryl and Lipiodol.

According to the authors' own experience, an antegrade approach via deep dorsal penile vein may be technically challenging but is preferential compared to a retrograde transfemoral access providing a straighter access route for catherization of especially periprostatic or internal pudendal venous leaks. Surgical exposure of the deep dorsal penile vein may be beneficial to enable venous access prior to transcatheter embolization in selected patients, e.g., in patients with deep dorsal venous hypoplasia.

## Results

A recent metanalysis identified 212 patients with veno-occlusive disease (9). Thereof, in 71 patients endovascular treatment was performed after surgical exposure of the deep dorsal vein, 126 were treated percutaneously without surgical exposure, and 13 were not further specified. Of all 212 patients, 109 (51.9%) were treated with N-butyl-2-cyanoacrylate and 94 (44.8%) were treated using a combination of embolic materials such as ethanol, sclerosant, coils, and gelfoam. Seven patients were exclusively treated with ethanol. In most of the patients the deep dorsal vein and periprostatic veins were embolized (69.5%) and other deep dorsal vein collaterals were embolized in 30.5% of patients.

### Outcomes

Technical success rates vary between 86 and 97% (Box 4). In one study technical failure was specified as failed access of the periprostatic veins via femoral access (18). In another study the deep dorsal vein could not be punctured in two patients either due to venous hypoplasia or venous alteration following previously attempted surgical ligation.

Box 4 **Outcomes of endovascular treatment of venogenic erectile dysfunction.**

Technical success rates for endovascular treatment of veno-occlusive dysfunction are high varying between 86 and 97%.

The overall clinical success rate for endovascular treatment of venous leaks is 59.5% ranging from 21.9 to 100% including partial and full responses. For full response, meaning sufficient erection to perform intercourse without additional need for supportive vasoactive medications, success rates tend to be lower.

Complication rates of endovascular treatment are low (5.2%), comprising mainly minor complications. Major complications such as pulmonary embolism are rare (<1%).

The overall clinical success rate was 59.5% (range 21.9–100%). However, clinical outcomes were heterogeneously defined and reported throughout different studies. Reporting varies from partial response to full response, with full response defined as sufficient erection to perform intercourse (19). However, partial response was not further defined. As reported by Fernandez et al., clinical success rate was 43.5%. In another study a scale for erectile quantity was introduced ranging from E0 (no erection) to E5 (normal erection) (18). According to this scale 82.7% of patients demonstrated clinical improvement, thereof 30% of patients with normal erection. Bookstein et al. defined clinical success as the ability to perform sexual intercourse and reported a success rate of 30.8% (20). Kutlu et al. reported a clinical success rate of 21.9% defining success as the ability to have sexual intercourse supported by vasoactive medication 1 year post treatment (21). Peskircioglu et al. observed clinical success in 68.8% of patients meaning successful restoration of erectile function. Rebonato et al. utilized both Doppler study and IIEF-6 questionnaire for follow-up (mean 13.3 months) (7). Clinical success was found in 72.2% of patients in their study. Yasumoto et al. reported clinical success in all patients (100%), but a strict definition was not yet provided (22). Schild et al. reported that 40 out of 58 patients (69%) demonstrated clinical success being able to have sexual intercourse (23). However, 19 patients needed additional vasoactive medication.

However, long-term results are still pending and results for partial response appear to be more advantageous than those for full response.

### Complications

A recent meta-analysis found an overall complication rate of 5.2 % (11 of 212 patients) post endovascular therapy of veno-occlusive disease (9). Thereof, two complications were classified as major complication and nine were classified as minor complication. Both patients with major complication demonstrated symptomatic pulmonary embolism during endovascular treatment (21). Minor complications included mild penis curvature (*n* = 2), mild perineal pain for several weeks (*n* = 2), asymptomatic pulmonary embolism (*n* = 1), partial subcutaneous reflux of N-butyl-2-cyanoacrylate associated with pain (*n* = 1), small subvesical hematoma (*n* = 1), epitheliolysis of penile glans due to allergic reaction to disinfectant (*n* = 1), and cough for 2 weeks post embolization (*n* = 1).

## Summary and Conclusion

In patients with erectile dysfunction, vasculogenic etiologies need to be considered if other causes such as neurogenic, psychogenic, and hormonal could be excluded (24). Vasculogenic etiologies include either arteriogenic or venogenic causes. Venogenic erectile dysfunction is due to veno-occlusive disease, also called “venous leak,” with incomplete relaxation of corporeal smooth muscle during arterial inflow and insufficient occlusion of venous outflow tracts. Dean et al. report that veno-occlusive disease may be due to multiple factors such as degenerative changes or injury of tunica albuginea, impaired relaxation of corporeal smooth muscles, venous shunting, and excessive adrenergic tone in anxious individuals (25).

In patients with veno-occlusive dysfunction, endovascular treatment with transcatheter embolization of venous leaks is performed to an increasing degree. The preferred access routes for transcatheter embolization is the deep dorsal vein with or without surgical exposure (7). Alternatively, access routes via common femoral veins have been used (18). Most notably, liquid embolic materials with low viscosity such as a combination of N-butyl-2-cyanoacrylate and ethiodized oil mixed in 1:1–1:3 ratios depending on quantity, size, and location of venous leaks are used for embolization and preferably causing venous inflammation, thrombosis, and fibrosis. Valsalva maneuver is required to avoid unintentional migration of glue with potential non-target embolization. If required, fibered coils may be used in advance in order to prevent expansion of glue through veins with fast outflow e.g., into femoral or iliac veins. Coils should be oversized up to 50% of the actual vein diameter to prevent inadvertent coil migration. Technical success rates range between 86 and 97%. Technical failures were due to failed direct deep dorsal vein puncture or failed access to periprostatic veins via femoral approach. In previous studies, complications rates were low (5.2%) consisting mainly of minor complications (9). However, symptomatic pulmonary embolism as formidable major complication occurred in two patients (<1%). Follow-up is performed using pharmacologic color Doppler exam combined with IIEF-6 questionnaire. The average clinical success rate in a recent meta-analysis was 59.5% ranging from 21.9 to 100% (9).

In conclusion, endovascular treatment is a promising approach in patients with erectile dysfunction due to veno-occlusive dysfunction which is increasingly utilized. Endovascular therapy with embolization of venous leaks is minimally invasive and may provide a safe alternative to surgical management. Further studies are needed to more adequately determine its role within the complex framework of manifold causes of erectile dysfunction.

## Author Contributions

HH: concept of manuscript, outline of topics, and scientific writing. ND: outline of topics and correction of manuscript. All authors contributed to the article and approved the submitted version.

## Conflict of Interest

The authors declare that the research was conducted in the absence of any commercial or financial relationships that could be construed as a potential conflict of interest. The handling Editor declared a past co-authorship with one of the authors ND.

## References

[1] NIH Consensus Conference. Impotence. NIH consensus development panel on impotence. Jama. (1993) 270:83–90. 10.1001/jama.270.1.83 8510302

[2] Nguyen HMT Gabrielson AT Hellstrom WJG. Erectile dysfunction in young men-a review of the prevalence and risk factors. Sex Med Rev. (2017) 5:508–20. 10.1016/j.sxmr.2017.05.004 28642047

[3] Rogers JH Goldstein I Kandzari DE Köhler TS Stinis CT Wagner PJ. Zotarolimus-eluting peripheral stents for the treatment of erectile dysfunction in subjects with suboptimal response to phosphodiesterase-5 inhibitors. J Am Coll Cardiol. (2012) 60:2618–27. 10.1016/j.jacc.2012.08.1016 23177300

[4] Dicks B Bastuba M Goldstein I. Penile revascularization–contemporary update. Asian J Androl. (2013) 15:5–9. 10.1038/aja.2012.146 23241636 PMC3739123

[5] Rogers JH Karimi H Kao J Link D Javidan J Yamasaki DS. Internal pudendal artery stenoses and erectile dysfunction: correlation with angiographic coronary artery disease. Catheter Cardiovasc Interv. (2010) 76:882–7. 10.1002/ccd.22646 20928837

[6] Lue TF. Erectile dysfunction. N Engl J Med. (2000) 342:1802–13. 10.1056/NEJM200006153422407 10853004

[7] Rebonato A Auci A Sanguinetti F Maiettini D Rossi M Brunese L. Embolization of the periprostatic venous plexus for erectile dysfunction resulting from venous leakage. J Vasc Interv Radiol. (2014) 25:866–72. 10.1016/j.jvir.2014.01.015 24613267

[8] Shafik A Shafik I El Sibai O Shafik AA. On the pathogenesis of penile venous leakage: role of the tunica albuginea. BMC Urol. (2007) 7:14. 10.1186/1471-2490-7-14 17803807 PMC1995196

[9] Doppalapudi SK Wajswol E Shukla PA Kolber MK Singh MK Kumar A. Endovascular therapy for vasculogenic erectile dysfunction: a systematic review and meta-analysis of arterial and venous therapies. J Vasc Interv Radiol. (2019) 30:1251–8. 10.1016/j.jvir.2019.01.024 31104902

[10] Diehm N Marggi S Ueki Y Schumacher D Keo HH Regli C. Endovascular therapy for erectile dysfunction-who benefits most? insights from a single-center experience. J Endovasc Ther. (2019) 26:181–90. 10.1177/1526602819829903 30741067

[11] Wespes E. Erectile dysfunction in the ageing man. Curr Opin Urol. (2000) 10:625–8. 10.1097/00042307-200011000-00016 11148737

[12] Rhoden EL Teloken C Sogari PR Vargas Souto CA The use of the simplified International Index of Erectile Function (IIEF-5) as a diagnostic tool to study the prevalence of erectile dysfunction. Int J Impot Res. (2002) 14:245–50. 10.1038/sj.ijir.3900859 12152112

[13] Kaufman JM Borges FD Fitch WP III Geller RA Gruber MB Hubbard JG. Evaluation of erectile dysfunction by dynamic infusion cavernosometry and cavernosography (DICC). Multi-institutional study. Urology. (1993) 41:445–51. 10.1016/0090-4295(93)90505-5 8488613

[14] Gratzke C Angulo J Chitaley K Dai YT Kim NN Paick JS. Anatomy, physiology, and pathophysiology of erectile dysfunction. J Sex Med. (2010) 7:445–75. 10.1111/j.1743-6109.2009.01624.x 20092448

[15] Ye T Li J Li L Yang L. Computed tomography cavernosography combined with volume rendering to observe venous leakage in young patients with erectile dysfunction. Br J Radiol. (2018) 91:20180118. 10.1259/bjr.20180118 30028186 PMC6475935

[16] Katzenwadel A Popken G Wetterauer U. Penile venous surgery for cavernosal venous leakage: long-term results and retrospective studies. Urol Int. (1993) 50:71–6. 10.1159/000282455 8460451

[17] Lewis RW. Venous surgery for impotence. Urol Clin North Am. (1988) 15:115–21. 3344555

[18] Aschenbach R Steiner T Kerl MJ Zangos S Basche S Vogl TJ. Endovascular embolisation therapy in men with erectile impotence due to veno-occlusive dysfunction. Eur J Radiol. (2013) 82:504–7. 10.1016/j.ejrad.2012.10.030 23219214

[19] Fernandez Arjona M Oteros R Zarca M Diaz Fernandez J Cortes I. Percutaneous embolization for erectile dysfunction due to venous leakage: prognostic factors for a good therapeutic result. Eur Urol. (2001) 39:15–9. 10.1159/000052406 11173933

[20] Bookstein JJ Lurie AL. Transluminal penile venoablation for impotence: a progress report. Cardiovasc Intervent Radiol. (1988) 11:253–60. 10.1007/BF02577012 3147140

[21] Kutlu R Soylu A Deep dorsal vein embolization with N-butyl-2-cyanoacrylate and lipiodol mixture in venogenic erectile dysfunction: early and late results. Radiol Oncol. (2009) 43:17–25. 10.2478/v10019-009-0011-2

[22] Yasumoto R Nishisaka N Sakakura T Kawano M Shindow K Takashima S Ethanol embolization for impotent patients with venous leakage: a new technique and initial results. Minim Invasive Ther Allied Technol. (1996) 5:564–6. 10.3109/13645709609152705

[23] Schild HH Müller SC Mildenberger P Strunk H Kaltenborn H Kersjes W Percutaneous penile venoablation for treatment of impotence. CardioVasc Interv Radiol. (1993) 16:280–6. 10.1007/BF02629158 8269423

[24] Bacon CG Mittleman MA Kawachi I Giovannucci E Glasser DB Rimm EB. A prospective study of risk factors for erectile dysfunction. J Urol. (2006) 176:217–21. 10.1016/S0022-5347(06)00589-1 16753404

[25] Dean RC Lue TF. Physiology of penile erection and pathophysiology of erectile dysfunction. Urol Clin North Am. (2005) 32:379–95. 10.1016/j.ucl.2005.08.007 16291031 PMC1351051

